# Effectiveness of online integrative trans-diagnostic treatment on internet addiction and high-risk behaviors in female adolescents with borderline personality disorder with comorbid depressive disorder

**DOI:** 10.3389/fpsyt.2023.1291579

**Published:** 2024-01-05

**Authors:** Fahimeh Mohamadpour, Nurallah Mohammadi

**Affiliations:** Department of Clinical Psychology, Faculty of Educational Sciences and Psychology, Shiraz University, Shiraz, Iran

**Keywords:** online integrative trans-diagnostic treatment, internet addiction, high-risk behavior, borderline personality disorder, adolescents

## Abstract

**Background:**

Based on research, borderline personality disorder is associated with many behavioral and emotional problems, including Internet addiction and high-risk behaviors. On the other hand, integrative trans-diagnostic treatment, by targeting trans-diagnostic factors in emotional pathology, is considered a suitable treatment for comorbid psychological pathologies. Also, since in adolescence, the opinion of others about oneself is more important, online therapy has the advantage of protecting them from the fear of stigma and shame in the face of others’ judgments. Therefore, the aim of the current research was to investigate the effectiveness of online integrative trans-diagnostic treatment on the internet addiction and high-risk behaviors in adolescents with borderline personality disorder.

**Methods:**

The current research was applied and quasi-experimental in a pre-test-post-test manner with a control group. The research sample included 40 female adolescents with borderline personality disorder who were randomly divided into two groups of 20 people, experimental and control groups. The criteria for entering the research included the age range of 12 to 18 years, female, confirmation of the disease by a psychologist, not receiving other psychological treatments in the last 3 months, and having a smartphone to participate in the online group. Substance abuse, any serious mental and physical illness/disorder that interferes with treatment, and failure to attend more than two sessions in therapy sessions were some of the exclusion criteria.The participants were tested on the scales of internet addiction and high-risk behaviors. Then, the treatment plan based on online integrative trans-diagnostic group therapy was presented to the participants of the experimental group online in 9 two-hour sessions. The control group was placed on the waiting list until the completion of the treatment sessions and the post-test implementation, and after the post-test implementation, they received the treatment. Then, their scores were measured, after completing the treatment, and a three-month follow-up period. Descriptive statistics and covariance test were used to analyze the data.

**Results:**

Patients showed a clear improvement in the severity of internet addiction and high-risk behavior symptoms. So that the scores of internet addiction and high-risk behaviors in the experimental group after receiving the treatment, as well as after a three-month follow-up period, were significantly reduced (*p* < 0.001).

**Conclusion:**

These results show that online integrative trans-diagnostic therapy can be an effective treatment option for reducing some symptoms of risky behaviors and internet addiction in adolescents with borderline personality disorder and should be studied further. It is noteworthy that the limitations of this study were the available sampling method, the use of a unisex sample of female patients, and the short-term follow-up period, which makes it difficult to generalize the results.

## Introduction

The topic of discussing the clinical diagnosis of borderline personality disorder (BPD) in adolescents has been around for a while. Nonetheless, there is a lot of focus on this disease right now, and research supports the diagnosis and treatment of BPD in adolescents (1). Adolescent borderline personality disorder (BPD) is now acknowledged as a specific disorder requiring intervention and treatment (2). BPD has an internalized aspect including dissociative identity disorder, paranoid thoughts, and chronic feelings of emptiness. It also has an emotional aspect, i.e., emotional instability, and an externalized aspect including impulsivity, suicide, self-injury behaviors, and unstable interpersonal relationships (3). World Health Organization’s extensive studies suggest that cluster B personality disorder patients are seven times more vulnerable to co-occurring mental disorders; so that, it has been estimated recently that 96% of BPD patients experience a type of functional or behavioral disorder in a period of their life (1, 4, 5).

The researches that have been conducted so far support the idea that some personality traits, such as borderline personality traits, are prominent in addictive behaviors, including Internet addiction (6) and various underlying and fundamental factors are common between these two disorders, among which impulsivity can be mentioned (7). Research has shown that people with borderline personality disorder use internet addiction as a way to cope with negative mood (8), reduce depressive symptoms (9) and reduce tension (10).

On the other hand, in the complex process of human growth and development, adolescence is more important, and in the meantime, research has shown that biological and social changes during adolescence are one of the prerequisites for the occurrence of various mental disorders including addictive behaviors (11). In this regard, the Adrenarche and Play systems, which are the biological prerequisites of adolescence, can easily interfere with each other and cause a disturbance in the balance of the brain and, as a result, make it more vulnerable to mental disorders (11).

The American Psychiatric Association has defined Internet addiction as a pattern of Internet use that leads to functional impairment and is accompanied by unpleasant internal states over a two-month period. To diagnose it, it has presented seven criteria (at least three criteria during 2 months): tolerance; withdrawal symptoms; The time of using the Internet will last longer than the person initially intends; persistent desire to control behavior; spending considerable time on Internet-related matters; Continued use despite being aware of its negative effects and reducing social, occupational and recreational activities as a result of using the Internet (12). In fact, Internet addiction is an impulse control disorder and a maladaptive pattern of Internet use that leads to significant discomfort or clinical disorder and creates psychological, educational, and occupational problems in a person’s life. The prevalence of this is higher in the age group of 15 to 19 years compared to other age groups (13). Recent studies have shown that the prevalence of Internet addiction among young people, in different societies and cultures, has a wide variation between 1.6 and 30%, and the rapid increase in the number of Internet users has also increased the prevalence of Internet addiction (13).

Risky behavior is also another variable that is associated with symptoms of borderline personality disorder. Research has shown that various types of risky behaviors and risk-taking are common in adolescents with borderline personality disorder (14). Risky behaviors can be defined as behaviors that will have unfortunate consequences for the individual, family, and society. Risky behavior is anything which may put ourselves or others at risk of physical, mental, emotional harm or abuse. These behaviors are more prevalent especially in adolescence and youth and include a wide range of behaviors such as theft, improper nutrition, risky sexual relations, dangerous driving and similar behaviors (15).

Considering the comorbidity and common features of borderline personality disorder with Internet addiction and tendency to risky behaviors, it seems necessary to choose a suitable treatment with the aim of controlling the common underlying symptoms between them. Among the psychotherapy approaches, trans-diagnostic treatment offers many advantages, which make it a perfect choice for measuring the improvement of common symptoms (16). The Unified Protocol for Trans-diagnostic Treatment proposed by Barlow et al. addresses a series of common trans-diagnostic factors that play an effective role in emotional disorders (16). Therefore, this protocol is designed and prepared to treat unipolar mood disorder and anxiety disorder patients as well as other emotional disorders, and can compensate the lack of pathology and comorbidity of different emotional disorders (16). Trans-diagnostic treatment is a suitable alternative treatment, where it is not possible to make homogenous groups (17). This unified protocol focuses on three factors, i.e., “cognitions,” “body sensations,” and “emotional behaviors.” These three factors are interrelated dynamically and each one of them plays an important role in an emotional experience (16).

Systematic reviews suggest that the unified trans-diagnostic therapy can contribute to the improvement of mental health, especially in a wide range of anxiety and depression disorders (17). Generally, effectiveness of this protocol in treatment of emotional disorders has been confirmed by different randomized control trials, which have been reflected in recent systematic reviews (18, 19). Grossman & Ehrenreich-May showed that trans-diagnostic treatment can reduce anxiety and intense emotional moods in adolescents suffering emotional disorders (20). Sandin et al. also showed that this treatment can reduce symptoms of anxiety, depression, anxiety sensitivity, emotional avoidance, phobia disorder, panic disorder, inclusive anxiety, and major depression in adolescents (21).

In mental health clinics, BPD is a common disorder in adolescents, and 10% of patients, nearly 50% of inpatients, and more than 80% of adolescents who have attempted suicide are estimated to suffer this disorder (4). Prevalence of this disorder at medium level is estimated about 9% in boys and about 13% in girls in adolescence ages (22). In addition, recent studies have emphasized that after the corona pandemic, women are more vulnerable to mental disorders and their consequences (23). Generally, different effective face to face treatment methods are available for abnormal behaviors (24). However, in different societies, there are different limitations to access the respective specialists. Such limitations include believing that the treatment is not warranted or effective, stigma, shame, bad experience of previous mental healthcare providers, and financial problems (25). Such factors are more common among the adolescents, who care more about the others’ opinion (26). This shows the importance of appropriate control and treatment, especially using online methods, for borderline personality disorder and related symptoms, especially behaviors in the adolescent age group. Accordingly, the present study aims to investigate the effectiveness of the online unified trans-diagnostic treatment approach in reducing internet addiction and high-risk behaviors in adolescents suffering borderline personality disorder.

### Purpose of study

The hypotheses of this research include the following:

1. online integrative trans-diagnostic treatment reduces internet addiction in female adolescents suffering borderline personality disorder.
2. online integrative trans-diagnostic treatment reduces high-risk behaviors in female adolescents suffering borderline personality disorder.

### Research method

The current research was applied research and quasi-experimental in the pre-test-post-test method with a control group.

### Participants

The statistical population in the present study was adolescent girls suffering from borderline personality disorder with comorbid depressive disorder living in Shiraz in 2022–2023, whose disease was diagnosed by a psychiatrist/clinical psychologist. In order to achieve reliable results in experimental designs, the presence of at least 15 people in each group is recommended (27). Therefore, among the statistical population of the research, 40 people were selected as the sample size and using the available sampling method from the statistical population. This method is a non-probability sampling method whose criterion is access to sample people. Available sampling involves the use of participants who are suitable for the research (28). Since the participants of the current study include people with a specific disorder and also, the participants must be willing to participate in therapy sessions, so the best option for initial sampling from the statistical population was to use this method. In the following, in order to be in the conditions of the experimental research, the participants were randomly (using the simple random method and receiving the code) placed in two control and experimental groups.

**Figure 1:**
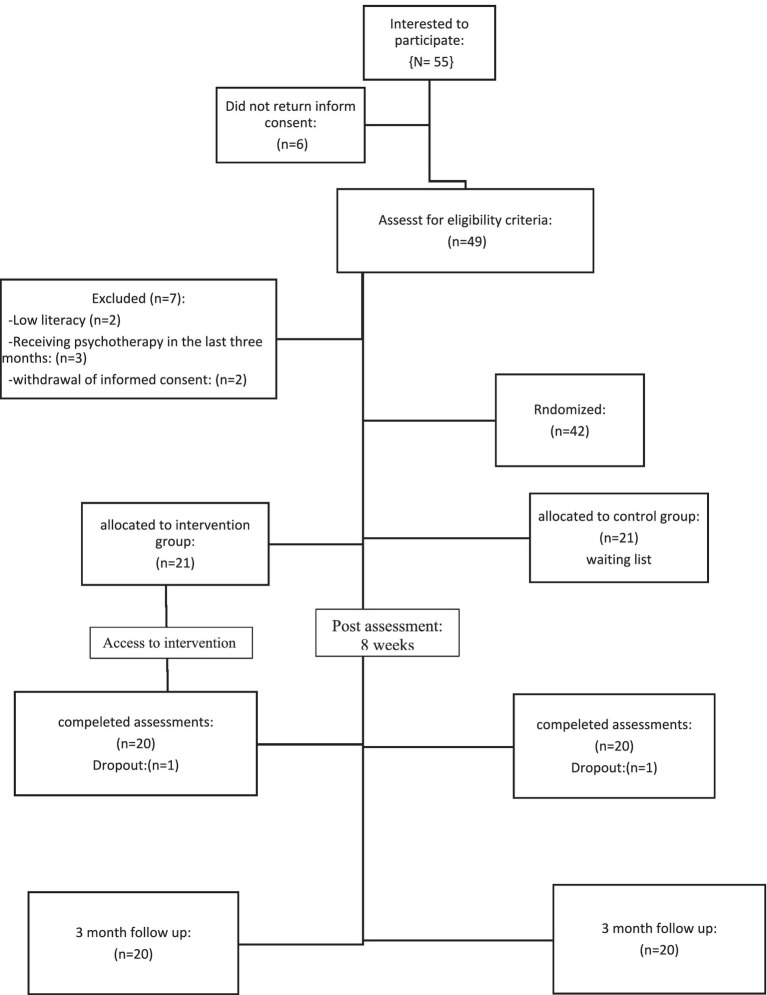
Participant flow.

The criteria for entering the research included the age range of 12 to 18 years, female, confirmation of the disorder by a psychologist, comorbid major depressive disorder with therapist diagnosis, not receiving other psychological treatments during the last 3 months, and having a smartphone in order to participate in the online group. Reluctance to participate in psychotherapy sessions, having psychotic disorders based on psychological examination, substance abuse, mental disability, any serious medical conditions interfering with treatment and absence of more than two sessions in therapy sessions were also the criteria for exiting the study. The flowchart of the participants is presented in Figure 1 at the end of the text.

### Procedure

The participants were randomly divided into two groups of 20 people, experimental and control. First, the participants were tested on the borderline personality disorder scales for children and adolescents and the score in the upper quartile of this scale was the final basis for selecting people. Also, they completed the scales of Internet addiction and high-risk behaviors. Control variables in this study included gender, age (12 to 18 years) and having a comorbid depressive disorder. Then, the treatment plan based on online integrative therapy was presented to the experimental group participants in 8 two-hour sessions and the control group was placed on the waiting list until the completion of the treatment sessions and the post-test implementation, and after the post-test implementation, they received the treatment. In order to achieve fidelity to the treatment, a pilot implementation was carried out before the implementation of the research. All treatment steps were carried out completely and accurately, based on the protocol presented in the research. Also, a supervisor who is proficient in this therapeutic approach, continuously monitored the therapist’s work process. After completing the treatment sessions of both groups, as well as after a three-month follow-up period, the participants were re-tested and the results were analyzed.

### Statistical analysis

Group differences in demographic data were tested with independent samples t-test for continuous variable (age) and chi-square test for nominal variables (socioeconomic status). To analyze the present results, a linear model was used by predicting the scores after the intervention, controlling the scores before the intervention as a covariate. So, the differences in the results of treatment implementation were evaluated based on one-way analysis of variance (ANCOVA; To compare one dependent variable in two control and experimental groups), considering treatment conditions as an inter-group factor. This approach includes the use of the covariance structure in the statistical control of hidden confounding variables, using the pre-test effect as the co-variate, and therefore, shows the best information indicators (29).

In order to achieve reliable results in experimental designs, the presence of at least 15 people in each group is recommended (27). Based on this, the current sample size was considered 15 people for each group.The evaluation of intra-group and inter-group changes due to the implementation of the treatment was compared in the control and experimental groups, and the follow-up evaluation was analyzed only for the intervention group and based on the post scores. Within- and betweengroup effect sizes (Cohen’s d) were calculated based on estimated means and the pooled standard deviation from the observed means. Richards and Richardson report an averaged effect size of Cohen’s d of 0.78 for guided interventions and of 0.36 for unguided interventions (30). Because guidance was used when requested, which could be considered between unguided and fully guided, a medium effect size of 0.50 was aimed. An error level of α 0.05 was also considered as a significance threshold (31). All analyses were performed in SPSS version 25.

### Research tools

#### Borderline personality disorder scale for children and adolescents

This scale is a 24-item self-assessment tool designed to assess borderline personality traits in children aged 12 to 17 years (32). The questions are scored as a five-degree Likert scale, from 1 (never) to 5 (always). The adolescents were asked to score their feelings about themselves and others. Higher scores reflect sever borderline personality symptoms and lower scores reflect minimal symptoms (33). Internal consistency of this scale was 75% (32). Psychometrical specifications of this questionnaire were studied for the first time in Iran by Zargar et al. (34). Validity of this scale was verified using three methods, i.e., Confirmatory Factor Analysis, Concurrent Validity, and Criterion Validity; and reliability of the scale was verified using Cronbach’s Alpha Correlation Coefficient. In this study, Cronbach’s alpha is reported 0.84 for the overall scale.

#### Internet addiction scale

The Yang Internet Addiction Questionnaire (IAT) has twenty questions, and the respondent must answer each question on a six-point Likert scale including: never, rarely, sometimes, often, and always, ranging from zero to five. The range of scores of this test is from 0 to 100, with a score of 0 to 39 representing the average user. 40 to 69 indicates mild internet addiction and 70 to 100 indicates severe internet addiction. Also, Cronbach’s alpha of this scale in Sweden and Korea is more than 0.9 and in the validity and reliability of this questionnaire in Iran, Cronbach’s alpha was 0.88 (35).

#### Iranian adolescent risk-taking scale (IARS)

This scale was developed by Zadehmohammadi et al. and contains 38 questions that measure 7 subscales. The questions are scored on a 5-point Likert scale from completely disagree (1 point) to completely agree (5 points). The reliability coefficient of this scale using Cronbach’s alpha is reported as 0.94, which indicates its good reliability (36).

### Integrative trans-diagnostic treatment

Barlow et al.’s trans-diagnostic treatment has a different structure than the well-known cognitive-behavioral treatments. The latest version of this therapeutic approach is based on “therapeutic heading” instead of “therapeutic sessions.” Each treatment chapter can take between one and several sessions, and based on the type of disorder, one chapter can take more time than other chapters. The recommended number of sessions, in general, is between 8 and 12 sessions and the suggested time of each session is between 50 and 60 min (16). Table 1 contains the goals and content of the meetings used in this research.

**Table 1:** Integrated meta-diagnostic treatment protocol.

Sessions	The aim of sessions/Content and techniques
Module 1	Increasing motivation to participate in treatment/Enhancing self-efficacy and belief in one’s ability to successfully achieve change/ Principles and techniques of motivational interviewing
Module 2	Psychological training and search for emotional experience/Psychological training based on the nature and recognition of emotions, the main components of emotional experience and the concept of learned responses.
Module 3	Emotional awareness training/Learning to identify how to react to emotions and manage positive and negative emotions Mindfulness technique - emotional induction technique - paying attention to the here and now
Module 4	Cognitive assessment and reassessment/The role of cognitive maladaptive automatic evaluations in creating and maintaining emotional experiences, investigating cognitive distortions, methods of correcting maladaptive thinking Discussion technique and cognitive reasoning
Module 5	Emotional avoidance and behaviors caused by emotion/ Emphasis on the behavioral components of emotional experience and familiarity with different patterns of emotional avoidance and how to change the current patterns of emotional responses Discussion technique and psychological training
Module 6	Awareness of physical feelings and their tolerance/Identifying the role of physical feelings in thoughts and behavior and their mutual influence and increasing tolerance towards these feelings Innate exposure technique or visceral exposure
Module 7	Endogenous and situation-based emotional exposure/Providing the logic of dealing with emotion and emotional arousal, Psychoeducational techniques, gradual exposure
Module 8	Prevention of relapse/An overview of treatment concepts and discussion about the improvement and progress of the patient, identification of ways to continue the possible results of the treatment and possible future problems Discussion technique

## Results

The research sample included 40 female adolescents suffering from borderline personality disorder and depression. The average age of the participants in this research was 15.39 with a standard deviation of 2.14, and they were randomly assigned to two control and experimental groups. Student’s t-test was used to measure the equality of two groups. The results showed that there is no significant difference between the two groups in terms of average age (*p* = 0.563; t28 = 0.461). In the present sample, all participants were single and in high school. Also, in order to measure the equality of two groups in socio-economic status, chi-square test was used. The results showed that there is no significant difference between the two groups in terms of this variable (Chi-square test = 1.219; *p* = 0.549). In Table 2, the descriptive statistics of the research variables are presented separately for the control and experimental groups.

**Table 2:** Mean and standard deviation of research variables in two experimental and control groups.

Variable	Group	Pre-test		Post-test		Follow up	
		Mean	S.D	Mean	S.D	Mean	S.D
Borderline personality disorder	Experiment	92.69	5.88	84.95	7.65	85.52	10.23
	Control	91.27	6.13	95.89	9.44	94.61	11.16
Internet Addiction	Experimental	111.61	10.74	87.41	14.12	85.32	15.48
	Control	113.52	11.24	114.98	13.56	113.67	16.27
High-Risk behaviors	Experimental	69.76	10.71	58.37	11.96	55.23	9.61
	Control	70.03	10.28	72.19	13.44	76.82	14.28

In order to implement the research hypotheses, Kolmogorov–Smirnov test was first performed to check the normality of the data and Leven’s test was also performed to check the equality of variances. Due to the fact that these two tests were not significant in any of the groups, therefore, the assumption of normality of data and equality of variances was maintained for all variables. Tables 3, 4 show the results of the Leven and Kolmogorov–Smirnov tests.

**Table 3:** Variance equality test.

Test	Variable	*F*	df1	df2	Sig.
Leven	Borderline personality disorder	1.204	4	26	0.310
	Internet addiction	1.382	4	36	0.291
	High-Risk behaviors	1.582	4	36	0.323

**Table 4:** Normality of the data test.

Test	Variable	*F*	df1	df2	Sig.
Kolmogorov–Smirnov	Borderline personality disorder	0.590	4	36	0.787
	Internet Addiction	0.564	4	36	0.761
	High-Risk behaviors	0.583	4	36	0.789

In examining the first hypothesis, the scores of borderline personality disorder, internet addiction and high-risk behaviors in the post-test were used as dependent variables and group (control, experimental) as independent variables, and their scores in the pre-test were used as covariate variables. Table 5 shows the effectiveness of the treatment on the post-test scores.

**Table 5:** The results of the covariance analysis of the difference in the average scores of the post-test in the experimental and control groups.

Variable	The source of variance	df	Mean of squares	*F*	*p*	Effect size
Borderline personality disorder	Group	1	1080.246	12.08	0/108	__
	Error	37	990.168	_		
Internet Addiction	Group	1	564.421	117.02	<0.001^*^	0.68
	Error	37	32.167	_		
High-risk behaviors	Group	1	376.584	58.416	<0.001^*^	0.62
	Error	37	37.267	_		

^*^p < 0.001.

As shown in Table 5, there is a significant difference between the groups in the scores of internet addiction and high-risk behaviors in the post-test (*F* = 117.02, *p* < 0.001; *F* = 58.416, *p* < 0.001). This means that the scores of this scale in the post-test in the experimental group are significantly different from the control group and the effectiveness of treatment on these two variables was 68 and 62%, respectively.

Also, the scores of the participants after a three-month follow-up period were measured using the analysis of covariance test, the results of which can be seen in Table 6.

**Table 6:** The results of the covariance analysis of the difference in mean follow-up scores in the experimental and control groups with pre-test control.

Variable	df	Mean of squares	*F*	*p*
Internet Addiction	27	587.268	122.397	<0.001^*^
Risky behaviors	27	395.672	61.025	<0.001^*^

^*^p < 0.001.

As can be seen in Table 6, the effects of treatment on the variables of internet addiction and high-risk behaviors remained after a 3-month follow-up period (*p* < 0.001).

## Discussion

The purpose of this study was to investigate the effect of the integrative trans-diagnostic treatment approach on internet addiction and high-risk behaviors in adolescents suffering borderline personality disorder. According to the findings, during ten treatment sessions, internet addiction scores have decreased significantly. The findings obtained in this research are in line with other researches about the effectiveness of this therapeutic approach in reducing various emotional and behavioral disorders (17, 18, 20). Considering the fact that excessive effort to control the emotional experience in a negative cycle can lead to an increase in the initial excitement, more unsuccessful attempts to avoid and, as a result, maintaining the uncomfortable situation, the first skill proposed in this protocol is being aware of and accepting the excitement. Other central topics used in this therapeutic approach include awareness of thoughts and cognition and helping to increase cognitive flexibility, facing external situations and using alternative strategies, facing bodily sensations and accepting and tolerating them (16). Negative emotions, such as depression, depend on cognition and behavior, and in this approach, regulating the emotions is the fundament of regulating cognitions and behaviors of a person. Additionally, accepting and being aware of emotions, without judging them, is another necessary skill for controlling emotions, which is also discussed in this approach (37).

In this method, management and regulation of emotions are emphasized that people with internet addiction have paid less attention to. On the one hand, Training to deal with negative emotions and cognitive reconstruction has caused a cognitive re-evaluation in adolescents suffering from internet addiction, and on the other hand, by reducing emotional suppression, it has removed the need for this group to express their emotions correctly. The result of this change in emotions and thoughts will be a behavioral change in the adolescent, which has already shown itself in the form of high-risk behavior and Internet addiction.

Another finding of this research indicated a decrease in the score of high-risk behaviors after receiving treatment with an integrative trans-diagnostic approach. This finding is also in line with the research conducted in this field (19, 21). In explaining this finding, it can be said that the integrative meta-diagnostic approach targets emotions and thus helps people to respond to them in a more adaptive way while facing their emotions. Based on this research logic, trans-diagnostic treatments have been designed and used to treat a wide range of disorders. Therefore, active participation in treatment, by trying to reduce subjectively experienced intolerable states, can help reduce the high risk behaviors in all categories of mental disorders. In other words, one of the reasons that can increase mental pressure in a stressful situation is intolerance and non-acceptance of negative emotions. One of the main goals in this therapeutic approach is to accept emotions and negative events as a part of human life. This type of treatment focuses on the adaptive and effective nature of emotions in general and without identifying a specific disorder, and therefore, it has shown supportive results in similar disorders. In addition, the main common points mentioned in emotional disorders include research evidence related to the high degree of concordance and diagnostic overlap and the generalization of therapeutic responses in these disorders (16). According to Barlow’s approach, primary emotions have an adaptive nature, but these secondary reactions along with judgment to these emotions make them unbearable and maintain the negative cycle of emotions. Reactions that are critical and judgmental and based on information that is not relevant to the present (16). By targeting people’s emotions and helping people to regulate their emotions, this therapy helps people learn to increase their tolerance for emotions, especially negative emotions, which is actually the underlying problem in people who engage in high-risk behaviors (38). Furthermore, another explanation that can be stated in this direction is the emphasis of integrated trans-diagnostic treatment on learning new and more adaptive behaviors in response to emotions, which actually, by teaching self-control of thoughts, reduces the person’s involvement in the negative cycle of self-harm in response to the excitement of the experience, and replacing them with more adaptive behaviors.

This awareness about positive abilities and teaching how to use these abilities correctly can ultimately increase the flexibility of adolescents in choosing efficient and appropriate activities and solutions in stressful situations. In fact, the adolescent’s power of choice in doing different activities changes from focusing on negative activities to paying attention to abilities that have been hidden until now. By simply replacing positive behaviors and capabilities, this can reduce attention to risky and harmful activities and, as a result, increase the adaptation of adolescents in stressful situations. On the other hand, paying attention to self-confidence and self-esteem in adolescents increases their daring skills, and on this basis, adolescents can stand stronger against the temptation of their friends and peers in dangerous situations. Therefore, this treatment approach, from several different dimensions, can come into action and help the adolescent to reduce high-risk behaviors and internet addiction as a behavioral disorder.

In addition, empirical evidence and strong theoretical foundations indicate common factors in the fields of symptomology, psychological, physiological and social etiology in various emotional disorders. Also, the high comorbidity in mental disorders creates significant problems for conceptualization and treatment and challenges the treatment, including the difficulty of conducting therapeutic exercises, evaluating progress, and focusing on a problem at a particular time. In addition, evidence-based treatments often have common elements such as cognitive restructuring, which leads to repetition with different treatment goals (39). These common factors show the need to pay attention to treatment with a trans-diagnostic approach, especially in comorbid disorders (16).

## Conclusion

In general, as mentioned, in borderline personality disorder, different emotional, cognitive and behavioral dimensions are involved (3). The focus of this research was on some of the behavioral disorders related to this group, including Internet addiction and high-risk behaviors in female adolescents. Overall, integrative meta-diagnostic treatment has been able to help improve behavioral symptoms in adolescents with borderline personality disorder by affecting symptoms of internet addiction and high-risk behaviors. Therefore, the current research has shown that the intervention based on the integrative trans-diagnostic approach can effectively help regulate emotions in adolescents with borderline personality disorder and replace maladaptive strategies of emotion regulation in the vicious cycle of emotional and psychological arousal of borderline personality disorder (including internet addiction and high-risk behaviors), to act as an effective treatment. In addition to this, the results of this research indicated that the use of treatment based on an integrative meta-diagnostic approach, in terms of time and cost savings, efficiency and effectiveness of implementation compared to other treatments that emphasize a specific structure can be a suitable option especially for signs and symptoms of comorbid disorders.

The meta-diagnostic approach to diagnosis and treatment has many practical advantages that can be considered a powerful option for measuring the improvement of common symptoms (16). Barlow et al.’s integrative transdiagnostic treatment protocol targets the set of common and transdiagnostic factors that cause emotional disorders, and therefore, it is designed and formulated for people with unipolar mood disorders and anxiety disorders with the ability to be applied to other emotional disorders as well. It can fill the gap of pathology and coexistence of different emotional disorders (16). Transdiagnostic treatment is a good option for treatment when homogeneous groups cannot be formed. Also, this treatment is considered the best option for patients whose symptoms do not complete any of the diagnostic criteria (17). Due to this, in the field of clinical psychology, this protocol can be used for the professional control and management of mental crises, as well as at the policy level for the training of healthcare personnel in similar situations.

It is worth mentioning that the implementation of treatment online has a lot to do with the type of treatment approach and exercises used in it. In this way, the techniques that can be implemented in the form of discussion or do not require extensive physical activities, are better applicable in online therapy. In the implementation of online therapy based on compassion, this therapeutic approach follows the same basic framework and only in some cases, more explanations are needed for the patient. In addition, the exercises used in it should be presented in a way that can be understood and implemented by the patients. Another fundamental difference in online implementation compared to face-to-face therapy is the greater emphasis on the principle of confidentiality, especially in group implementation. In other points, online implementation is almost similar to face-to-face therapy, and therefore, it can be expected that by following these points and strictly implementing the treatment protocol, the result will be similar to face-to-face therapy, as was also observed in the present study.

### Limitations and future research

While applying the findings of the present study, one of the limitations of this study was the available sampling method, the use of a unisex sample of female patients, and the short-term follow-up period, which makes it difficult to generalize the results. It is noteworthy that the current statistical procedure does not control for the effect of attrition (dropouts), and that future studies should consider following an Intention to Treat (ITT) analysis. In addition, it should be noted that the use of online methods to treat Internet addiction must be carefully considered precisely because of the risk of encouraging what one would like to reduce.

It is suggested that in order to develop the findings of the current research, male samples should be used. Furthermore, in order to evaluate the stability of grades, long-term follow-up periods should be measured. In addition, using a mixed sample size, examining the effectiveness of treatment on other age groups, and considering other mental disorders with high comorbidity with borderline personality disorder will greatly contribute to the development of knowledge in this field.

## Data availability statement

The original contributions presented in the study are included in the article/supplementary material, further inquiries can be directed to the corresponding authors.

## Ethics statement

The studies involving humans were approved by Shiraz University Ethical Committee under approved N: IR.US.PSYEDU.REC.1402.027. The studies were conducted in accordance with the local legislation and institutional requirements. Written informed consent for participation in this study was provided by the participants' legal guardians/next of kin.

## Author contributions

FM: Writing – original draft, Writing – review & editing. NM: Writing – original draft, Writing – review & editing.
